# Between Eagle and Dragon: Affective representations of the United States and China in South Korean Media

**DOI:** 10.1371/journal.pone.0352240

**Published:** 2026-06-22

**Authors:** Seungwoo Han

**Affiliations:** Kyonggi University, Suwon, South Korea; Southwest University, CHINA

## Abstract

Emotions are closely implicated in how states are represented and interpreted. This study examines the affective dimensions of South Korean media portrayals of the United States and China from 1992 to 2025, focusing on variation in evaluative tone and emotional intensity. Drawing on theories of affective framing and media agenda-setting, the analysis traces long-term patterns in valence and arousal using a large corpus of newspaper articles and a computational text analysis approach. The results indicate a persistent asymmetry. Coverage of the United States remains relatively stable, with moderately positive evaluations and low levels of emotional intensity, whereas portrayals of China become more negative and more affectively charged over time. These differences appear as sustained patterns rather than isolated fluctuations and are consistent with broader features of South Korea’s geopolitical context, including alliance relations, historical memory, and media environments. By documenting these patterns, this study contributes to the analysis of affect in media discourse and provides a framework for examining how major powers are represented in national contexts over time.

## Introduction

Emotions, far from being peripheral to international politics, constitute foundational elements in the construction of global political meaning. As Hutchison and Bleiker [1] propose, affect plays a critical role in shaping how actors perceive, interpret, and respond to international events. In recent years, affective polarization has emerged as a salient feature of international media discourse, particularly in East Asia [2–6], where geopolitical rivalry, contested historical memory, and enduring alliance structures converge to produce emotionally charged narratives [7–10]. Nowhere are these dynamics more evident than in South Korea, where media portrayals of the United States and China exhibit marked asymmetries in emotional tone, patterns that mirror deeper institutional, ideological, and strategic alignments. While the U.S.–ROK alliance continues to serve as the cornerstone of South Korea’s security architecture and normative orientation, China’s regional ascent has generated public ambivalence, elite anxiety, and heightened affective discourse across the political spectrum.

The present study examines how South Korean media portray the United States and China along two affective dimensions: valence, referring to the evaluative tone of coverage, and arousal, capturing the intensity of emotional expression. It focuses on the domestic affective environment within which foreign policy discourse is articulated. Drawing on theories of agenda-setting, framing, and affective intelligence, the analysis considers how patterns of emotional tone are reflected in media representations of international actors. Methodologically, this study analyzes a large corpus of newspaper articles published between 1992, when diplomatic relations between South Korea and China were established, and 2025. Using a large language model (LLM), it measures the emotional tone of each article to trace long-term patterns in valence and arousal across media coverage of the two countries.

The findings indicate a persistent affective asymmetry. Coverage of the United States is characterized by moderate, low-arousal evaluations, consistent with the routinized and institutionally embedded nature of the bilateral alliance. In contrast, portrayals of China have become increasingly negative and more emotionally intense over time.

The results further suggest that representations of major powers in South Korean media are structured not only by strategic considerations but also by the emotional logics embedded in media discourse. These patterns form part of the broader interpretive environment within which foreign policy debates take place.

This study makes three contributions. First, it applies a LLM to the analysis of affective content in media discourse, providing a scalable approach to measuring valence and arousal. Second, it documents systematic differences in how the United States and China are represented in South Korean media and situates these patterns in relation to alliance structures, historical memory, and ideological media environments. Third, by tracing these patterns over an extended period, it shows that affective differences in media coverage are sustained over time rather than confined to specific events.

In the subsequent sections, this study situates South Korean media portrayals of the United States and China within broader theoretical frameworks of alliance politics and affective framing. It then analyzes the emotional tone of media coverage directed toward each country, drawing on longitudinal trends in valence and arousal. The analysis concludes by considering how these affective asymmetries relate to broader discussions of South Korea’s geopolitical identity and positioning.

## The affective politics of U.S.–China Portrayals in South Korean Media

### Alliance politics and asymmetrical power dynamics

The affective framing of the United States and China in South Korean media cannot be disentangled from the structural imperatives of the U.S.–ROK alliance. Institutionalized in the aftermath of the Korean War, this alliance forms the bedrock of South Korea’s national security architecture and continues to shape its strategic imagination [11]. As a junior partner in this asymmetrical relationship, South Korea remains dependent on American military support and normative leadership, a dynamic that reinforces the United States’ centrality within South Korea’s geopolitical worldview [12,13]. Positioned by Washington as a frontline state in its “freedom frontier” strategy across the Indo-Pacific, South Korea’s role is both strategic and symbolic [14]. This hierarchical configuration not only informs foreign policy decisions but also exerts a formative influence on domestic media narratives [11,15].

In such a context, South Korean mass media tend to reproduce alliance-friendly narratives, depicting the United States in a favorable, stabilizing light [11,15]. Conservative outlets, in particular, emphasize the strategic indispensability of the alliance, often portraying the United States as a reliable patron and guarantor of regional order. This tendency aligns with broader patterns observed in alliance-dependent states, wherein positive affect toward the hegemon helps reinforce elite consensus and sustain public legitimacy for the alliance [16].

Indeed, studies have shown that South Korean media dedicate disproportionate coverage to alliance affairs relative to their American counterparts, reflecting the embeddedness of the alliance in public discourse and political life [11,15]. Moreover, the mediatization of foreign policy in democratic contexts such as South Korea means that political elites anticipate and respond to media framing, often crafting foreign policy statements in ways that resonate with alliance-supportive narratives. As a result, the structural imperative of alliance maintenance operates not only at the level of diplomatic institutions but also through the symbolic and emotional economy of mass communication.

### Historical memory, strategic tension, and the construction of threat

Whereas the United States is positioned as a strategic ally, China is increasingly represented as a geopolitical rival and civilizational Other. This divergence reflects a complex interplay of historical memory, strategic anxiety, and national identity. South Korea’s historical relationship with China is marked by deep ambivalence. On one hand, China has long been a cultural reference point and historical partner; on the other, it has been a source of subordination and interference, from the tributary system to the Chinese military intervention during the Korean War [17–19]. These historical layers of asymmetry continue to inform contemporary perceptions, rendering China a particularly sensitive object of national discourse [7].

In recent years, a series of bilateral tensions have contributed to the consolidation of what might be termed a “China threat” frame in South Korean media. These include disputes over historical interpretation (such as China’s Northeast Project) [20], cultural appropriation controversies (involving kimchi and traditional attire) [21], and high-profile geopolitical flashpoints such as China’s retaliation against South Korea’s THAAD (Terminal High Altitude Area Defense) deployment [22,23]. In each instance, media coverage has framed China as either violating Korean sovereignty or acting with arrogance and coercion. This process is intensified by South Korea’s proximity to China and the asymmetry of power between the two countries, which amplifies anxieties about Korea’s autonomy and status.

Media representations of China are thus often saturated with high-arousal emotional cues, portraying China as a revisionist power whose actions are not merely undesirable but threatening [10,24]. These affective framings are particularly salient in coverage of issues involving economic coercion [22], environmental harms [25], China’s historical ties with North Korea [26], or pandemic diplomacy [27], where China is frequently cast as irresponsible or predatory [9,28]. The resulting narratives do not emerge in a vacuum but resonate with prevailing public sentiment.

In addition to security and historical factors, economic competition constitutes an important background condition shaping media representations [29]. Technonationalism, which treats technological capability as central to national security and economic sovereignty [30–33], has re-emerged across major political economies [34]. Recent policy developments, including the United States’ CHIPS and Science Act [35], China’s “Made in China 2025” strategy [36–38], and the European Union’s push for digital sovereignty [39], reflect a broader shift toward state-led techno-industrial competition. This rivalry extends beyond material competition over supply chains and standards to encompass symbolic contestation over technological leadership [40]. Within this context, the expansion of Chinese firms in sectors such as telecommunications, digital platforms, and advanced manufacturing has intensified competitive pressures on Korean industries, providing a structural backdrop for increasingly negative and emotionally charged media portrayals of China [6].

Survey data consistently demonstrate that South Koreans tend to hold unfavorable views of China, in contrast to the generally positive and reliable image attributed to the United States [41–43]. This divergence in public perception is consistent with a dynamic in which media representations and public attitudes may interact over time, contributing to the persistence of affective asymmetries in public discourse. Over time, such framings contribute to the symbolic construction of China as a threatening Other, shaping not only media discourse but the broader emotional architecture of geopolitical perception.

### Conservative elite alignment, partisan media, and ideological reinforcement

Media systems are embedded within broader structures of political and economic power. Far from neutral conveyors of information, they function as strategic institutions that reproduce dominant ideological formations. The political economy of media [44,45] and structuralist approaches such as Althusser’s [46] concept of ideological state apparatuses (ISAs) offer foundational frameworks for understanding how media reinforce prevailing class relations through routinized communication practices.

A central insight from this tradition is that news production is shaped by structural constraints, particularly ownership concentration, commercial revenue imperatives, and professional norms [47,48]. Where media ownership is concentrated among conglomerates with close ties to political and economic elites, journalistic content tends to reflect elite consensus. Curran and Seaton [49] describe this as a systemic bias favoring narratives that stabilize existing power relations. Commercial pressures exacerbate this effect, as advertising dependence discourages coverage that challenges corporate interests or alienates affluent audiences [50].

These dynamics are especially visible in South Korea’s ideologically polarized media landscape. Media ownership is concentrated among conglomerates aligned with conservative elites [51], shaping editorial priorities and foreign policy framing [52,53]. Major conservative dailies such as *Chosun Ilbo* and *JoongAng Ilbo* consistently promote pro-U.S. narratives and adopt hawkish stances toward authoritarian regimes like China, often invoking Cold War-era frames that equate communism with threat.

This ideological alignment has historical roots in South Korea’s authoritarian period. During the regimes of Park Chung Hee and Chun Doo-hwan, conservative media played a central role in disseminating anti-communist narratives under state censorship regimes, including the Basic Press Act of 1980 [52,54,55]. While democratization curtailed direct censorship, institutional legacies remain [56,57]. As Althusser [46] emphasizes, ideology endures not only through coercion but through the habitual normalization of dominant worldviews embedded in institutional practice.

Progressive media in South Korea, while frequently critical of American unilateralism and military overreach [58], do not necessarily adopt a more favorable stance toward China. Rather, outlets such as *The Hankyoreh* often frame foreign policy through the lens of Korean nationalism and sovereign autonomy, which can lead to critical depictions of Chinese behavior, particularly when it is perceived as encroaching upon Korea’s national dignity or security [8,59,60]. Instances such as China’s economic retaliation following South Korea’s deployment of the THAAD missile defense system elicited cross-ideological consensus [61], with both conservative and progressive media expressing unified opposition. In addition, during the COVID-19 pandemic, both conservative and progressive media articulated negative sentiment toward China, especially in relation to issues of sovereignty and national risk [62]. *The Hankyoreh*, in particular, has portrayed China as a civilizational “Other,” reinforcing a broader nationalist narrative that positions Korea as a “model state” surpassing China in governance and global standing [8].

These patterns suggest that critical portrayals of China are not confined to conservative media but are widespread across the ideological spectrum, albeit grounded in differing normative frameworks. While conservative outlets tend to emphasize strategic and ideological threats, progressive media are more likely to foreground infringements on national autonomy and cultural integrity. This convergence contributes to a media environment in which negative coverage of China is not only normalized but pervasive. In contrast, the United States, despite occasional criticism, remains comparatively insulated from sustained negative scrutiny, reflecting its embedded role as a security partner and normative reference point. The intersection of elite interests, ideological orientations, and institutional media practices thus reinforces affective asymmetries in South Korean public discourse, shaping the emotional and symbolic architecture of international representation.

### Framing, affect, and geopolitical identity

Beyond institutional configurations, media effects theories offer essential analytical tools for examining how political meanings are constructed and sustained through discourse. Agenda-setting theory, introduced by McCombs and Shaw [63], posits that the media influence public priorities by determining which issues are granted visibility. This foundational insight was expanded by second-level agenda-setting theory [63], which emphasizes that media not only shape the *salience* of issues but also influence how they are interpreted, by accentuating particular attributes, evaluative frames, or moral dimensions of political actors and events.

Framing theory provides a deeper account of how media structure public meaning through selective representation. According to Entman [64], framing entails the strategic emphasis of certain elements of perceived reality in order to define problems, assign causality, render moral judgments, and propose solutions. These frames are embedded within broader political and institutional contexts and function to reproduce dominant ideological assumptions [65–67]. Framing, in this sense, operates as a mechanism that aligns public perception with institutional interests and normative orders. This logic extends beyond domestic politics into the international sphere [68–71].

South Korean media discourse exemplifies these dynamics in the representation of international relations. The United States is routinely framed as a strategic ally and normative referent, reflecting the legacy of the U.S.–ROK alliance and broader ideological identification with liberal democracy [15]. In contrast, China is frequently portrayed as a source of instability, coercion, or normative challenge, particularly in the context of regional security concerns, historical disputes, and authoritarian governance [72]. These portrayals are not merely informational but are affectively charged, relying on emotionally resonant language and imagery to shape public orientations and consolidate geopolitical narratives.

The integration of affect into media and communication theory has further illuminated the role of emotion in shaping political cognition and behavior. Drawing on affect theory and political psychology, scholars argue that emotional responses are central to how media messages are processed and internalized [48,73]. Affect operates not as a secondary feature of cognition but as a foundational mode of meaning-making in political communication.

Within this framework, two key emotional dimensions—valence (the positivity or negativity of tone) and arousal (the intensity of emotional activation)—are particularly salient for understanding media effects [74,75]. High-arousal emotions such as fear and anger have been found to increase message retention, heighten perceptions of threat, and catalyze political engagement, albeit in often polarizing ways [76].

Affective Intelligence Theory [73] provides a theoretical basis for distinguishing these effects. While enthusiasm tends to reinforce habitual partisan behavior, anxiety disrupts routine and facilitates learning, and anger mobilizes political defense. These affective responses are often elicited through media frames that portray political actors and issues in emotionally provocative terms. As such, affective framing is not peripheral but central to the dynamics of political communication [77].

In the South Korean context, these dynamics manifest in the divergent emotional framing of the United States and China. Positive portrayals of the United States reinforce a national self-image grounded in democratic values and Western alignment. Conversely, negative and high-arousal depictions of China contribute to the symbolic construction of an externalized Other, against which notions of national identity, sovereignty, and legitimacy are asserted. Through these mechanisms, affective framing forms part of the broader emotional architecture through which geopolitical identity is represented and interpreted.

### Mediatization and foreign policy discourse

In South Korea, the mediatization of foreign policy has transformed the media from a passive conduit of state messaging into an active agent in constructing international political meaning. Rather than merely transmitting official positions, media discourse forms part of the environment within which foreign policy agendas are articulated, by selectively amplifying issues and framing them in emotionally resonant terms. This is particularly relevant in democratic contexts, where media narratives operate as one of several channels through which public sentiment and elite discourse interact, shaping how policy options are publicly framed and debated.

The South Korean case illustrates how affective media framings, especially those invoking national security, identity, and historical memory, are associated with patterns that reflect symbolic hierarchies within the international order. Persistent portrayals of the United States as a stabilizing ally and normative reference point, and of China as a coercive and emotionally charged threat, are not reducible to editorial bias or partisanship. These asymmetries are embedded in a broader matrix of alliance structures, ideological dispositions, and historical legacies, and are consistent with patterns observed in South Korea’s strategic culture and geopolitical self-conception.

These framings may contribute to a recursive dynamic. Emotionally charged representations can become part of the environment within which public perceptions and elite discourse interact over time. Foreign policy discourse thus unfolds within a mediated field structured by affective cues, where meaning is produced not only through institutional deliberation but also through the emotional logics of mass communication.

These dynamics highlight the role of media in the emotional framing of international politics. The privileging of the United States and the marginalization of China in South Korean media are consistent with broader strategic, institutional, and symbolic alignments that shape how foreign policy is imagined and debated.

## Methodology

### Research design

To examine affective patterns in South Korean media coverage of the United States and China, this study employs a computational content analysis approach grounded in political communication theory. Central to this approach is the use of a large language model (LLM) to analyze full-text newspaper articles, enabling the automated classification of emotional tone along two theoretically informed affective dimensions: valence, referring to the evaluative positivity or negativity of the coverage, and arousal, which denotes the intensity or emotional activation conveyed.

All data were collected from publicly accessible news articles via NAVER, a major news aggregator and search engine in South Korea. The data collection process relied on standard search functionality and did not involve any circumvention of access restrictions or protected content. The collection and analysis procedures were conducted in accordance with the terms of use of the data source.

The affective categories are drawn from foundational theories in political psychology and media studies, which emphasize the central role of emotion in shaping political communication, persuasion, and mobilization [74,75]. Valence operates as a signal of approval or disapproval, thereby influencing political attitudes, while arousal conditions the level of psychological engagement and action-readiness. Together, these dimensions provide an integrated analytical framework for understanding how emotional framing in media discourse contributes to the reproduction and contestation of political power.

To analyze emotional tone in Korean-language news coverage, this study employs LLaMA-3 (specifically LLaMA-3-8B, hereafter LLaMA-3), an open-source LLM, to classify articles along the affective dimension: valence (positive–negative evaluation), rather than relying on a Korean sentiment lexicon-based encoder model such as BERT [51]. LLaMA-3 shares key architectural features with proprietary models such as GPT-4 but offers distinct advantages for academic research, including transparent implementation, reproducibility, and computational efficiency, making it particularly well-suited for longitudinal, large-scale text analysis [78]. Recent applications of LLM-based sentiment analysis in Korean-language corpora provide additional support for the feasibility of this approach [6,79,80].

The model was implemented in a Python-based environment using structured Korean-language prompts and applied in a zero-shot setting, meaning it was not fine-tuned on domain-specific labeled data. This approach allowed for efficient and consistent inference across the full corpus of about 400,000 news articles.

While LLaMA-3 is trained primarily on English-language data, approximately 5% of its pretraining corpus consists of high-quality non-English content across more than 30 languages, including Korean [81]. Recent benchmarking studies show that models of this class can reliably extract affective features even in languages with relatively limited representation [82–84].

**Fig 1:**
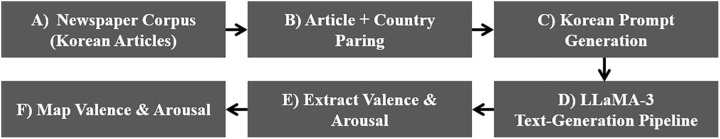
Schematic Diagram: Valence and Arousal classification pipeline.

Fig 1 presents a schematic overview of the analytical pipeline, outlining the sequential procedures employed in the classification of emotional tone. The process begins with the collection of full-text newspaper articles referencing either the United States or China, spanning the period from 1992 to 2025. Each article is categorized by country and paired with a structured Korean-language prompt designed to elicit an evaluative response from the language model. The LLaMA-3 model is applied to classify each article along two theoretically informed affective dimensions: valence and arousal. The model’s textual outputs are subsequently parsed and transformed into continuous numerical scores for both dimensions. Further information on prompt design and scoring methodology is provided in S1 Table from Supporting information. Details regarding specific modelling procedures and estimation techniques are presented within the respective analytical sections.

### Llama-3 sentiment analysis

This study employs LLaMA-3, an open-source LLM accessed via the Hugging Face Transformers API, to classify the emotional tone (valence and arousal) of full-text Korean newspaper articles. The classification is implemented in a Google Colab Pro environment using the LLaMA-3-8B model, with the transformers, torch, and accelerate libraries in Python.

GPU hardware typically included NVIDIA T4 or A100 units with 16–40 GB of VRAM and approximately 32 GB of RAM. Due to memory constraints, inference was performed in small batches (1–2 articles per pass), using mixed-precision (fp16) and 8-bit quantization via the bitsandbytes library to reduce memory load. To ensure consistency and reproducibility, all preprocessing steps, such as tokenization, prompt formatting, and truncation to 512 tokens, were standardized. Caching mechanisms were used to avoid redundant processing across sessions.

To operationalize affective classification, the model was provided with a structured prompt that defined evaluative categories in accordance with established frameworks in political communication research [85]. The model was instructed to assign scores from 0 to 1 based on the article’s overall affective tone. Valence was scaled continuously from 0 (very negative) to 1 (very positive), reflecting evaluative polarity. Arousal was coded in parallel to capture emotional intensity, ranging from 0 (minimal activation) to 1 (high emotional activation).

To assess the validity of the LLM-based classifications, a random subset of 1,000 articles was selected for human annotation. Three independent coders, including the author, rated each text on three-point scales—negative, neutral, or positive for valence, and low, medium, or high for arousal [86]. Coders were given general instructions for evaluating valence and arousal, and a brief discussion was conducted prior to coding to align interpretations across raters. Disagreements were resolved by majority rule. For comparison with human annotations, model-generated continuous scores were converted into categorical labels using evenly spaced thresholds: [0–0.33] as negative, [0.34–0.66] as neutral, and [0.67–1.00] as positive. The thresholds were defined to align with the three-category structure of the human annotations, allowing for direct comparison between model outputs and coder evaluations [86].

Agreement between model and human classifications reached 78 percent for valence and 67 percent for arousal, indicating stronger correspondence in evaluative polarity than in emotional intensity. Per-class performance metrics provide additional detail. For valence, the average precision, recall, and F1 scores across the three categories were 0.75, 0.72, and 0.73, respectively. For arousal, the corresponding averages were 0.67, 0.63, and 0.65, reflecting moderate consistency typical of affective annotation in cross-lingual contexts. Inter-coder reliability among human annotators, measured with Fleiss’s κ, was 0.82 for valence (substantial agreement) and 0.69 for arousal (moderate agreement). These results are consistent with prior findings that arousal tends to elicit greater interpretive variability than valence [86–88].

### Data

To construct the dataset, a custom Python-based web scraping framework was developed to collect Korean-language newspaper articles referencing the keywords, “United States” and “China”. The data collection was conducted through NAVER, South Korea’s largest news aggregator and search engine, which accounted for approximately 55 percent of national market share as of 2024. For each country, this study retrieved 500 articles per month from January 1992 to February 2025, yielding a total of 398,000 articles (see S3 Table in Supporting information for scraping process).

The decision to implement a fixed-quota sampling design was motivated by analytic priorities: by holding constant the number of articles per firm and time unit, the analysis focuses exclusively on variation in affective content, without conflation by fluctuations in coverage volume. It allows for a clearer identification of framing trends, controlling for differences in visibility that may arise from external events.

Articles were selected based on NAVER’s search rankings and were not filtered for semantic duplication. This reflects a deliberate choice to preserve the structural characteristics of the Korean media environment, where content syndication and duplication are prevalent [89,90]. Syndicated stories and wire content often appear across multiple outlets with minimal variation, reinforcing affective narratives through repetition and cross-platform exposure. Rather than treating such duplication as noise, this study incorporates it as a meaningful feature of the media system.

The scope of collection was not restricted to diplomatic coverage but included all articles referencing the United States and China across political, economic, cultural, and social domains. This inclusive sampling strategy was designed to capture the broader emotional landscape of media discourse surrounding the two countries over time.

## Results of analysis

The results are presented in two parts. Analysis 1 provides a descriptive overview of monthly trends in valence and arousal, revealing asymmetries in the emotional framing of the United States and China across time. Analysis 2 extends this by testing whether these differences constitute statistically significant patterns of divergence, using time-interacted regression models to estimate long-term trends in evaluative tone and emotional intensity.

### Analysis 1: Sentiment and arousal patterns in media coverage of the United States and China

**Fig 2:**
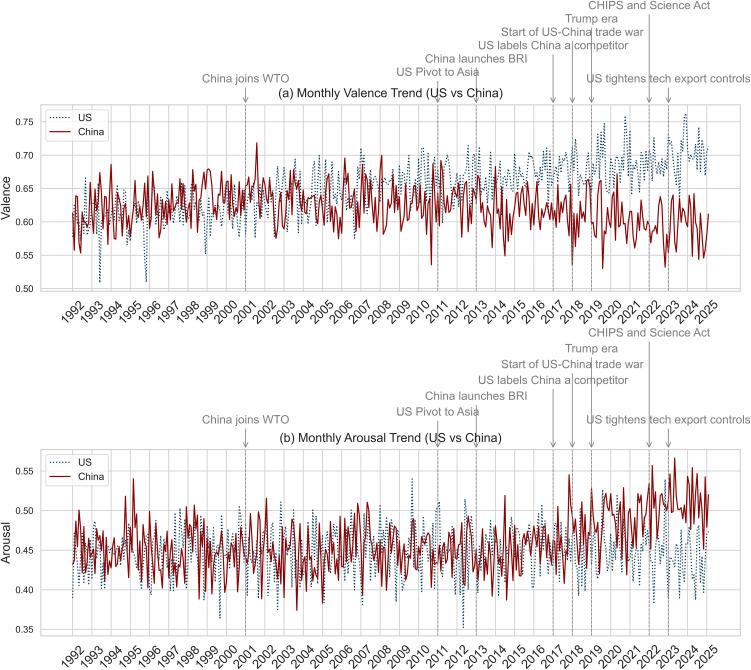
Mass Media Trends of Valence and Arousal (1992-2025).

To evaluate how South Korean mass media have portrayed the United States and China over time, this study analyzes valence and arousal scores derived from a longitudinal dataset of full-text newspaper articles published between January 1992 and February 2025. Fig 2 displays monthly time-series trends for each country, with valence capturing the overall evaluative tone of coverage, ranging from 0 (very negative) to 1 (very positive), and arousal reflecting emotional intensity, ranging from 0 (low emotional intensity) to 1 (highly emotive).

The time-series plot in Fig 2 reveals a clear asymmetry in the affective framing of the two countries. Coverage of the United States exhibits a high degree of temporal stability, with consistently moderate to positive valence and relatively subdued arousal. This continuity suggests that the United States is portrayed in the South Korean media as a familiar and largely uncontroversial actor, likely reflecting the institutionalized nature of the U.S.–ROK alliance and the routinization of bilateral engagement. Although there are occasional fluctuations, they do not appear to deviate significantly from a long-term pattern of affective normalization.

By contrast, the portrayal of China is characterized by relatively more dynamic and volatile trends across both dimensions. Valence scores for China begin at a similarly positive level in the 1990s but exhibit a gradual decline starting in the mid-2010s, becoming more pronounced after 2018 and continuing into the early 2020s. At the same time, arousal scores for China increase steadily over the same period, culminating in heightened levels of emotional intensity in the most recent years. This combination of declining sentiment and rising arousal suggests an evolving media narrative in which China is not only viewed less favorably but also discussed in increasingly urgent or emotive terms.

Importantly, these shifts correspond closely to key moments in the trajectory of U.S.–China relations, including the United States’ pivot to Asia in 2011, China’s launch of the Belt and Road Initiative in 2013, the Trump administration’s designation of China as a strategic competitor in 2017, and the onset of the U.S.–China trade war in 2018. More recent developments such as the passage of the CHIPS and Science Act in 2022 and the tightening of U.S. export controls in 2023 appear to coincide with the most marked shifts in China-related sentiment and arousal. Although South Korean media are not themselves direct participants in this bilateral rivalry, the results suggest that their affective framing of China is increasingly filtered through a U.S.-centric geopolitical lens. This pattern is consistent with a broader shift in how Chinese power is discursively constructed in the South Korean public sphere.

**Fig 3:**
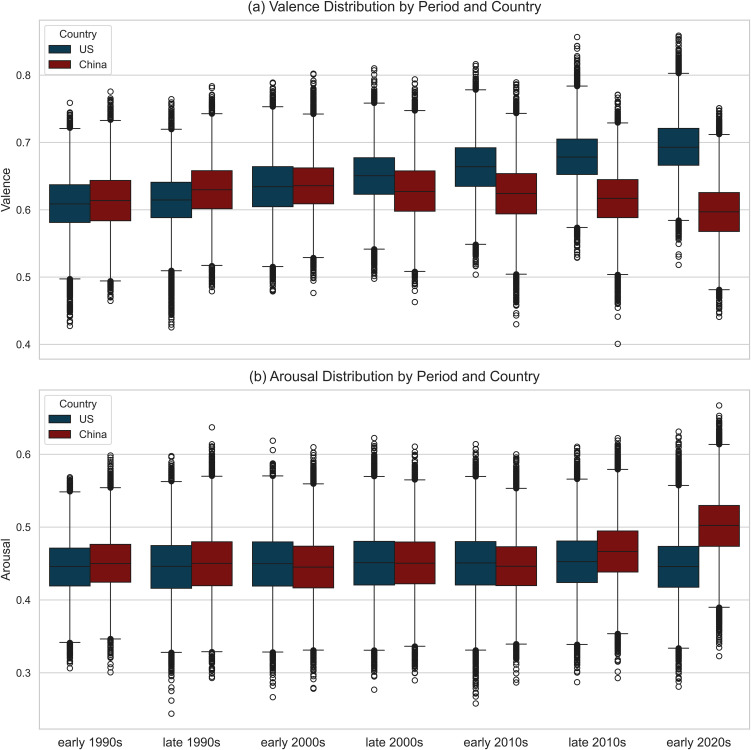
Valence and Arousal Distribution (Box Plot) by Period and Country.

To contextualize these temporal trends within broader historical periods, Fig 3 presents box plots of valence and arousal scores, segmented into the early and late halves of each decade. This format enables a comparative assessment of medium-term shifts in affective framing across distinct geopolitical periods. During the early 1990s, sentiment toward both the United States and China was broadly positive, and emotional arousal remained low. These patterns reflect the optimism of the post–Cold War era and the initial normalization of diplomatic ties between South Korea and China.

This general tone of positivity persisted through the late 1990s, although a modest uptick in arousal scores for China suggests growing media attentiveness to its evolving regional role. The early 2000s maintained overall favorable sentiment toward both countries, yet the late 2000s marked the onset of divergence. While valence scores remained relatively stable, arousal levels associated with China began to rise—indicating increased emotional salience in media representations.

This divergence became more pronounced during the 2010s. In the early part of the decade, valence toward China began to decline, while arousal levels continued to climb. These trends accelerated in the late 2010s amid growing geopolitical tensions, including trade disputes, and security issues. By the early 2020s, affective asymmetries between the two countries reached their peak: Chinese portrayals were marked by intensified emotional activation and increasingly negative evaluations, whereas coverage of the United States remained comparatively moderate in tone and consistent in affective stability.

### Analysis 2: Comparative dynamics in affective asymmetry

**Fig 4:**
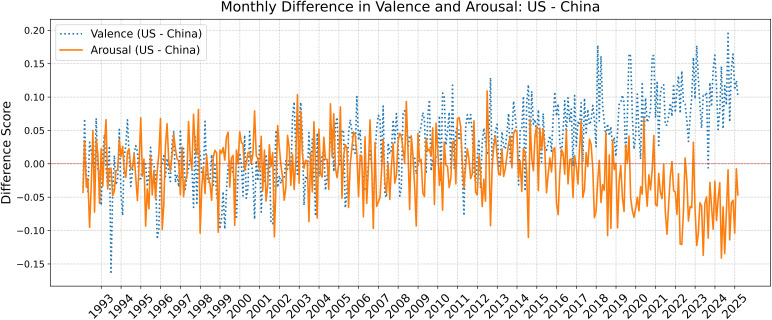
Monthly Difference in Valence and Arousal: U.S.-China.

Fig 4 visualizes the evolving emotional gap in South Korean media coverage of the United States and China by plotting the monthly difference in valence and arousal scores between the two countries. The blue line represents the difference in valence scores (U.S. valence – China valence), capturing the relative positivity of coverage. The orange line reflects the difference in arousal scores (U.S. arousal – China arousal), capturing the relative emotional intensity or affective charge.

An increasing blue line signifies that South Korean media are portraying the United States in increasingly more positive terms than China, that is, the *positive–negative gap* is widening in favor of the United States. This upward trend in valence difference suggests a growing evaluative asymmetry, in which China is being framed more negatively relative to the United States over time.

Conversely, a decreasing orange line (i.e., when the line dips downward) would indicate that the United States is being described in less emotionally intense terms compared to China, or that China-related content is becoming more affectively charged. In this context, the graph shows a decreasing orange line, particularly after the mid-2010s, indicating that China is increasingly portrayed in a more emotionally heightened and arousing manner than the United States.

To assess whether emotional tone in South Korean media coverage of the United States and China has diverged over time, the analysis estimates a regression model using monthly country-level averages of valence (or arousal) as the dependent variable. The model includes a binary indicator for China-related coverage (China), a continuous time variable (Time), and their interaction term (China × Time). Time is defined as the number of months since January 1992, coded from 0 through the end of the sample period in February 2025.

In this specification, the United States serves as the reference category. The coefficient on Time captures the temporal trend in valence or arousal for U.S.-related coverage, while the interaction term (China × Time) identifies whether China follows a distinct trajectory over time. This allows for a direct test of whether affective portrayals of China have diverged from those of the United States across the period of analysis.

Including a continuous time variable also accounts for broader shifts in the media environment, such as changes in journalistic practices, technological developments, or evolving geopolitical conditions, which may otherwise confound observed trends.

To address potential serial correlation and heteroskedasticity in the time-series structure, the model employs Newey–West heteroskedasticity and autocorrelation consistent (HAC) standard errors with a 12-month lag [91]. The analysis is based on 796 monthly observations (398 months × 2 countries). Given that the dependent variables are bounded between 0 and 1, linear models are used as approximations of average effects, particularly in the presence of interaction terms and conditional comparisons [92]. Accordingly, the estimates are interpreted as linear projections rather than structural probabilities. The model is specified as follows:


$$\begin{array}{c} {Valence\;(or\;Arousal)}_{it}=\beta_{\text{0}}+\beta_{\text{1}}\cdot {China}_{i}+\beta_{\text{2}}\cdot {Time}_{t}+\beta_{\text{3}}\cdot \left({China}_{i}\times {Time}_{t}\right)+\varepsilon_{it} \end{array}$$

As presented in Table 1, the valence model reveals a strong and consistent trend. The coefficient on time is positive and statistically significant (β = 0.0003, *p* < 0.001), indicating that U.S.-related sentiment has become more positive over the 1992–2025 period. The initial advantage of China in 1992, reflected in the positive China dummy (β = 0.0344, *p* < 0.001), has eroded over time. The interaction term is negative and highly significant (β = −0.0003, *p* < 0.001), confirming that valence in China-related coverage has declined relative to that of the United States. These results provide evidence that China is being framed increasingly negatively in South Korean media.

**Table 1:** Regression Estimates of Valence and Arousal Trends in U.S.–China Media Coverage (1992–2025).

DV	Intercept	China	Time	China × Time	DW stat
Valence	0.6011*** (0.003)	0.0344*** (0.006)	0.0003*** (1.06e-05)	−0.0003*** (2.29e-05)	2.019
Arousal	0.4464*** (0.002)	−0.0117* (0.005)	0.00001 (1.06e-05)	0.0001*** (2.71e-05)	2.032

*Note*: ***p < 0.001, **p < 0.01, *p < 0.05. Standard errors are Newey–West HAC, 12-month lag.

By contrast, the arousal model shows that while the U.S. trajectory has remained largely stable (non-significant time trend), China-related coverage has intensified in emotional charge over time. The interaction term is positive and significant (β = 0.0001, *p* < 0.001), indicating that China is increasingly portrayed in high-arousal, emotionally provocative terms. Although initial arousal levels for China were slightly lower than those for the United States (β = −0.0117, *p* = 0.028), the emotional intensity gap has since reversed and widened in China’s direction.

The Durbin–Watson statistics (2.019 for valence and 2.032 for arousal) are close to the ideal value of 2, suggesting no substantial evidence of serial correlation in the residuals. When considered alongside the application of Newey–West standard errors to correct for potential heteroskedasticity and autocorrelation, these diagnostics indicate that the regression estimates are reasonably robust.

These results confirm a dual pattern of affective divergence: the United States is represented with increasing positive valence and affective stability, while China is portrayed with growing negativity and heightened emotional intensity. As Fig 4 illustrates, the divergence in both valence and arousal scores becomes most pronounced from the mid-2010s onward, consistent with broader geopolitical developments and alliance dynamics discussed earlier in the study.

Additional robustness checks were conducted using alternative covariance specifications. The main results are consistent across models estimated with heteroskedasticity-robust (HC3) standard errors and those employing Newey–West HAC corrections with different lag lengths (6 and 12 months) in S4 Table. In both the valence and arousal models, the key interaction term (China × Time) remains stable in direction, magnitude, and statistical significance. While the precision of the main time trend in the arousal model decreases under HAC corrections, the substantive pattern of increasing emotional intensity in China-related coverage remains unchanged. These findings indicate that the results are robust to alternative assumptions about error structure and temporal dependence.

Collectively, the results presented in Analysis 1 and 2 suggest a gradual but significant transformation in the affective architecture of South Korean media discourse. The United States continues to be represented in affectively consistent terms, marked by moderate positivity and low emotional volatility. In contrast, portrayals of China have undergone a clear shift, becoming more negative in tone and more emotionally intense in framing. These findings highlight the salience of affective differences in media coverage and suggest that media discourse is associated with broader patterns in regional power dynamics.

## Conclusion

This study has examined the affective dimensions of South Korean media portrayals of the United States and China over a three-decade period, identifying a consistent asymmetry in emotional tone. Coverage of the United States is characterized by relatively stable and moderately positive evaluations with low levels of emotional intensity, whereas portrayals of China have become increasingly negative and more affectively charged, particularly since the mid-2010s. These patterns are not limited to discrete events but appear as sustained differences over time.

The results are consistent with an interpretation in which media representations are embedded in broader structural contexts, including alliance relations, historical memory, and ideological orientations. In this sense, the relative stability of U.S.-related coverage and the intensification of negative portrayals of China may reflect the positioning of these countries within South Korea’s geopolitical environment. At the same time, these findings should be interpreted as describing patterns in media discourse rather than as direct evidence of public opinion or foreign policy behavior. The analysis identifies systematic differences in media representations without making claims about their direct effects on attitudes or policy outcomes.

This distinction is important for interpreting the scope of the findings. Media discourse constitutes one component of a broader communicative environment in which perceptions and policy debates take place, but it does not map directly onto either public preferences or state behavior. The extent to which affective patterns in media coverage translate into measurable changes in attitudes or policy remains an open empirical question. Future research could address this more directly by linking media-based measures of affect with survey data or indicators of foreign policy positioning.

Before turning to the broader implications, several limitations of this study should be noted. First, the analysis does not incorporate variation in the ideological orientation of South Korea’s governing administrations, instead focusing on long-term affective patterns in media discourse. This design choice allows for a clearer identification of structural trends, but it also leaves aside a potentially important source of variation. In South Korea’s highly mediatized political environment, shifts in government, particularly between conservative and progressive administrations, have been shown to shape both foreign policy positioning [93] and the framing of international actors in domestic media [15]. As a result, the estimates presented here should be understood as capturing aggregate tendencies over time rather than administration-specific dynamics. Future research could examine how changes in governing ideology interact with media framing to produce more contingent patterns of affect.

Second, the measurement strategy relies on a LLM to classify emotional tone, which introduces its own set of constraints. While the absence of supervised training data avoids some forms of researcher-induced bias, it does not eliminate the possibility of systematic error, particularly in cases where distinctions in valence or arousal are subtle [94]. This issue is compounded by the application of a multilingual model to Korean-language texts. Emotional expression in Korean news discourse is often conveyed indirectly, through context-dependent phrasing or culturally embedded evaluative cues, which may not be fully captured in a zero-shot classification setting. The validation results suggest a reasonable correspondence with human coding, but the resulting measures are best interpreted as approximations of affect rather than precise indicators. In addition, the dataset is constructed from articles retrieved through NAVER’s search system. Because search rankings are shaped by algorithmic criteria such as relevance and user engagement, certain outlets or types of coverage are likely to be more visible than others. This feature can be understood as part of the contemporary media environment, in which exposure is structured by platform logics. At the same time, future research could employ alternative sampling strategies to assess how different data construction approaches may shape observed patterns.

Finally, although the analysis situates media portrayals within a broader geopolitical context, it does not directly model the economic dimensions that intersect with South Korea’s relations with the United States and China. The rise of Chinese technology firms during the 2010s, and their expansion into sectors such as telecommunications, digital platforms, and advanced manufacturing, has intensified competition with Korean firms in both domestic and global markets [36–38]. This evolving industrial rivalry likely forms part of the background against which media narratives have shifted, potentially contributing to more negative and emotionally heightened portrayals of China [6]. While economic competition is considered here as a contextual condition, incorporating more explicit measures of industrial structure or firm-level competition would allow future research to more precisely assess how economic and geopolitical factors jointly shape affective representations of international actors.

Within these limits, the findings point to a persistent divergence in how major international actors are represented in South Korean media. The relative stability of U.S.-related coverage and the increasing negativity and emotional intensity associated with China suggest that affective framing is structured in ways that align with broader geopolitical orientations. These patterns may shape the terms through which international relations are discussed, even if their direct effects on attitudes or policy cannot be inferred from the present analysis.

This study contributes to the literature in three respects. First, it documents long-term differences in affective media representations of major powers, showing that such patterns are sustained rather than episodic. Second, it demonstrates the use of LLMs for analyzing valence and arousal in large-scale text data, providing a scalable approach to the study of affect in political communication. Third, by situating media discourse within broader structural contexts, it highlights how affective representations may form part of the interpretive environment in which international politics is understood and debated.

## Supporting information

S1 Table Dual-Dimensional Emotion Classification of Korean News Articles Using LLaMA-3. (DOCX)

S2 Table Descriptive analysis. (DOCX)

S3 Table Python web scraping framework (Naver news). (DOCX)

S4 Table Robustness Checks Across Covariance Specifications. (DOCX)
