# Supplementary material for: Between Eagle and Dragon: Affective representations of the United States and China in South Korean Media
Source: PLoS One. 2026 Jun 22;21(6):e0352240. doi: 10.1371/journal.pone.0352240 (PMC13286215; doi:10.1371/journal.pone.0352240)
Supplement: S1 Table — (DOCX) [file pone.0352240.s001.docx]

**S1 Table.** Dual-Dimensional Emotion Classification of Korean News Articles Using LLaMA-3

| **# Step 0: Install required packages**  !pip install transformers accelerate bitsandbytes pandas tqdm --quiet  **# Step 1: Load the model with memory-efficient settings**  from transformers import AutoTokenizer, AutoModelForCausalLM, pipeline  import torch  model_name = "meta-llama/Llama-3-8b-hf"  # Load tokenizer  tokenizer = AutoTokenizer.from_pretrained(model_name)  # Load model with 8-bit quantization for Colab memory efficiency  model = AutoModelForCausalLM.from_pretrained(  model_name,  device_map="auto",  torch_dtype=torch.float16,  load_in_8bit=True  )  # Initialize generation pipeline  generator = pipeline(  "text-generation",  model=model,  tokenizer=tokenizer,  max_new_tokens=100,  do_sample=False  )  **#Step 2. Define Korean Prompt**  def build_prompt_korean_continuous(article_text, country_name):  return f"""  You are a political news sentiment analyst. Analyze the emotional attitude expressed in the following news article toward the country "{country_name}".  Article:  "{article_text.strip()}"  Tasks:  1. Valence: Quantify the overall evaluation of the country expressed in the article on a scale from 0 (very negative) to 1 (very positive).  2. Arousal: Quantify the intensity of emotional expression on a scale from 0 (little or no emotional intensity) to 1 (very strong emotional intensity).  Please respond in the following format:  Valence: [0.0 - 1.0]  Arousal: [0.0 - 1.0]  """  **#Step 3. Inference & Parcing Logic**  def parse_response_continuous(text):  valence, arousal = None, None  for line in text.split('\n'):  if 'Valence' in line:  try:  valence = float(line.split(":")[1].strip())  except:  valence = None  elif 'Arousal' in line:  try:  arousal = float(line.split(":")[1].strip())  except:  arousal = None  return valence, arousal  **#Step 4. Batch Processing Function**  from tqdm import tqdm  import pandas as pd  def classify_articles_continuous(df, article_col='article', counry_col='country'):  results = []  for _, row in tqdm(df.iterrows(), total=len(df)):  article = row[article_col]  country = row[country_col]  prompt = build_prompt_korean_continuous(article, country)  output = generator(prompt)[0]['generated_text']  valence, arousal = parse_response_continuous(output)    results.append({  'country': country,  'article': article,  'valence': valence,  'arousal': arousal  })  return pd.DataFrame(results)  **#Step 5. Load and Run**  df = pd.read_csv("full_articles.csv") # 144,000 articles  results_df = classify_articles(df)  # Save to disk  results_df.to_csv("llama3_sentiment_results.csv", index=False) |
| --- |
