# Supplementary material for: Between Eagle and Dragon: Affective representations of the United States and China in South Korean Media
Source: PLoS One. 2026 Jun 22;21(6):e0352240. doi: 10.1371/journal.pone.0352240 (PMC13286215; doi:10.1371/journal.pone.0352240)
Supplement: S2 Table — (DOCX) [file pone.0352240.s002.docx]

**S2 Table**. Descriptive analysis

|  | **N** | **Mean** | **Std dev** | **Min** | **Median** | **Max** |
| --- | --- | --- | --- | --- | --- | --- |
| Valence | 398000 | 0.636 | 0.049 | 0.401 | 0.63 | 0.858 |
| Arousal | 398000 | 0.453 | 0.044 | 0.243 | 0.453 | 0.667 |
