# Supplementary material for: Between Eagle and Dragon: Affective representations of the United States and China in South Korean Media
Source: PLoS One. 2026 Jun 22;21(6):e0352240. doi: 10.1371/journal.pone.0352240 (PMC13286215; doi:10.1371/journal.pone.0352240)
Supplement: S3 Table — (DOCX) [file pone.0352240.s003.docx]

**S3 Table.** Python web scraping framework (Naver news)

| from bs4 import BeautifulSoup  import requests  import openpyxl  from openpyxl import Workbook  import pandas as pd  from openpyxl.utils.dataframe import dataframe_to_rows  import time  import pyautogui  from docx import Document  from openpyxl.styles import Alignment  import datetime  import re  **# 1. Keywords and pages**  keyword = pyautogui.prompt("insert keyword")  lastpage = int(pyautogui.prompt("How many pages?"))  #create excel  wb = Workbook()  ws = wb.active  ws.column_dimensions['A'].width = 60  ws.column_dimensions['B'].width = 60  ws.column_dimensions['C'].width = 120  ws.column_dimensions['D'].width = 40  ws.column_dimensions['E'].width = 40  **# 2. Specify contents for scraping in Naver news**  row = 2  ws.append(['link','title', 'contents', 'date', 'company'])  page_num = 1  for i in range(1, lastpage*10,10):  print(f"{page_num}now scraping.==================================================")  response = requests.get(f"https://search.naver.com/search.naver?where=news&sm=tab_pge&query={keyword}&sort=0&photo=0&field=0&pd=3&ds=2012.01.01&de=2012.01.30&cluster_rank=22&mynews=0&office_type=0&office_section_code=0&news_office_checked=&nso=so:r,p:from20120101to20120130,a:all&start={i}")  html = response.text  soup = BeautifulSoup(html, 'html.parser')  articles = soup.select("div.info_group") # news text crol + f    **# 3. Collecting news**  for article in articles:  company = article.select_one("a.info.press") # press  links = article.select("a.info") # list    if len(links) >= 2: # if link 2 or more  url = links[1].attrs['href'] # extract href from second link  response = requests.get(url, headers = {'User-agent': 'Mozila/5.0'})  html = response.text  soup_sub = BeautifulSoup(html, 'html.parser')  # if entertainment news  if "entertain" in response.url: # add response.  if soup_sub.select_one(".end_tit") == None:  pass  else:  title = soup_sub.select_one(".end_tit")    if soup_sub.select_one("#articeBody") == None:  pass  else:  contents = soup_sub.select_one("#articeBody")    if soup_sub.select_one("div.article_info > span > em") == None:  pass  else:  date = soup_sub.select_one("div.article_info > span > em")    elif "sports" in response.url:  if soup.select_one("h4.title") == None:  pass  else:  title = soup.select_one("h4.title")    if soup.select_one("#newsEndContents") == None:  pass  else:  contents = soup.select_one("#newsEndContents")  dives = content.select("div") # erase unnecessary contents such as email address    for div in dives:  div.decompose()    paragraphs = content.select("p")  for p in paragraphs:  p.decompose()  if soup_sub.select_one("div.article_info > span > em") == None:  pass  else:  date = soup_sub.select_one("div.article_info > span > em")      else:  if soup_sub.select_one(".media_end_head_headline") == None:  pass  else:  title = soup_sub.select_one(".media_end_head_headline")    if soup_sub.select_one("#dic_area") == None:  pass  else:  contents = soup_sub.select_one("#dic_area")    if soup_sub.select_one("span.media_end_head_info_datestamp_time._ARTICLE_DATE_TIME") == None:  pass  else:  date = soup_sub.select_one("span.media_end_head_info_datestamp_time._ARTICLE_DATE_TIME")  # print("==========link==========\n", url)  # print("==========title==========\n", title.text.strip())  # print("==========text==========\n", contents.text.strip())  # print("==========date======= \n", date)  # print("=========outlet======= \n", company.text.strip())    # save in excel  ws[f'A{row}'] = url  ws[f'B{row}'] = title.text.strip()  ws[f'C{row}'] = contents.text.strip()  ws[f'D{row}'] = date.text.strip()[:7]  ws[f'E{row}'] = company.text.strip()    row = row + 1    time.sleep(0.5)      page_num = page_num + 1 |
| --- |
