# Supplementary material for: Between Eagle and Dragon: Affective representations of the United States and China in South Korean Media
Source: PLoS One. 2026 Jun 22;21(6):e0352240. doi: 10.1371/journal.pone.0352240 (PMC13286215; doi:10.1371/journal.pone.0352240)
Supplement: S4 Table — (DOCX) [file pone.0352240.s004.docx]

**S4 Table.** Robustness Checks Across Covariance Specifications

| (a) Valence | | | |
| --- | --- | --- | --- |
| Model | China × Time | Std. Error | p-value |
| HC3 (no autocorrelation correction) | -0.0003 | 1.16e-06 | <0.001 |
| HAC (lag = 6) | -0.0003 | 2.09e-05 | <0.001 |
| HAC (lag = 12) | -0.0003 | 2.29e-05 | <0.001 |
| (b) Arousal | | | |
| HC3 (no autocorrelation correction) | -0.0003 | 1.17e-06 | <0.001 |
| HAC (lag = 6) | -0.0003 | 2.29e-05 | <0.001 |
| HAC (lag = 12) | -0.0003 | 2.71e-05 | <0.001 |
